# RadB acts in homologous recombination in the archaeon *Haloferax volcanii*, consistent with a role as recombination mediator

**DOI:** 10.1016/j.dnarep.2017.04.005

**Published:** 2017-07

**Authors:** Kayleigh Wardell, Sam Haldenby, Nathan Jones, Susan Liddell, Greg H.P. Ngo, Thorsten Allers

**Affiliations:** a School of Life Sciences, University of Nottingham, Queen’s Medical Centre, Nottingham, UK; b School of Biosciences, University of Nottingham, Sutton Bonington, UK

**Keywords:** Homologous recombination, Archaea, RecA-family recombinase, Strand exchange, Recombination mediator

## Abstract

Homologous recombination plays a central role in the repair of double-strand DNA breaks, the restart of stalled replication forks and the generation of genetic diversity. Regulation of recombination is essential since defects can lead to genome instability and chromosomal rearrangements. Strand exchange is a key step of recombination – it is catalysed by RecA in bacteria, Rad51/Dmc1 in eukaryotes and RadA in archaea. RadB, a paralogue of RadA, is present in many archaeal species. RadB has previously been proposed to function as a recombination mediator, assisting in RadA-mediated strand exchange. In this study, we use the archaeon *Haloferax volcanii* to provide evidence to support this hypothesis. We show that RadB is required for efficient recombination and survival following treatment with DNA-damaging agents, and we identify two point mutations in *radA* that suppress the Δ*radB* phenotype. Analysis of these point mutations leads us to propose that the role of RadB is to act as a recombination mediator, which it does by inducing a conformational change in RadA and thereby promoting its polymerisation on DNA.

## 1 Introduction

Homologous recombination (HR) plays a central role in the repair of DNA double-strand breaks and the generation of genetic diversity in meiosis or conjugation – HR functions may also contribute to the restart of stalled DNA replication forks. Although HR is critical for cell viability, it can pose significant risks if improperly regulated. Suboptimal HR can result in inaccurate repair of DNA damage and accumulation of mutations. Conversely, excessive HR can result in DNA rearrangements. Several genetic diseases that are linked to increased cancer risk are associated with defects in HR regulation. These include Bloom’s syndrome and Werner’s syndrome, which are characterised by increased levels of recombination due to defective RecQ family helicases that function at different stages of HR [1], [2].

The central step of HR is strand exchange, which is catalysed by RecA-family recombinases: RecA in bacteria, Rad51/Dmc1 in eukaryotes and RadA in archaea. Deletion of recombinase genes leads to defects in HR and an increased sensitivity to DNA-damaging agents [3], [4]. The first stage of HR initiated at a DNA end is 5′-3′ end resection, which produces single-stranded DNA (ssDNA) onto which the recombinase protein polymerises. Activation of the recombinase is carried out by recombination mediators as described below. Nucleoprotein filaments consisting of recombinase and ssDNA then bind to double-strand DNA (dsDNA) molecules and search for a region of homology. When homology is found, the recombinases catalyse strand invasion and D-loop formation [5].

*In vivo*, ssDNA produced by end resection is coated with the single-strand DNA binding protein, termed SSB (in bacteria) or RPA (in eukaryotes and most archaea). The binding of SSB/RPA protects ssDNA from secondary structure formation and degradation, and is an important stage of HR. However, SSB/RPA poses a barrier to recombinase filament formation, since these proteins compete with recombinases for DNA binding.

Recombination mediators are a class of proteins required for efficient HR, which may assist in recombinase nucleoprotein filament formation by overcoming the inhibition imposed by SSB/RPA. They also play a role in stabilising nucleoprotein filaments. Deletion of mediator genes leads to defects in recombination and DNA repair. Examples of bacterial recombination mediators include Rec(F)OR, a complex that assists in the loading of RecA onto ssDNA [6], and RecX and DinI, which stabilise the RecA nucleoprotein filament [7], [8]; the bacterial Sms recombination modulator was recently shown to stimulate the branch migration phase of RecA-mediated strand transfer [9]. Eukaryotic recombination mediators include BRCA2 in humans, and Rad52 and Rad55-Rad57 heterodimer in yeast, all of which assist in the displacement of RPA and loading of Rad51 onto ssDNA [10], [11]. Rad55-Rad57 in yeast has also been shown to play a role in stabilising Rad51-DNA filaments from disassembly by the anti-recombinase Srs2 [12]. The balance between these two processes is thought to be a key regulatory step in controlling the initiation of HR. Recent work has shown that the Rad51 paralogue RFS-1 from *Caenorhabditis elegans* functions as a recombination mediator in combination with a partner protein, RIP-1 [13]. RFS-1/RIP-1 is proposed to stimulate HR by remodelling the Rad51 presynaptic filament into a more flexible structure that is less prone to disassembly by helicases.

Two archaeal recombination mediators have been identified, both are paralogues of RadA. SsoRal1 is found in *Sulfolobus solfataricus* and has been shown to stimulate RadA-mediated strand exchange *in vitro* by enhancing RadA binding to ssDNA [14]. RadB, which is found only in members of the phylum Euryarchaeota, has been proposed to function as a recombination mediator [15]. Genetic evidence has shown that deletion of *radB* increases the DNA damage sensitivity of *Haloferax volcanii* [16]. Furthermore, RadA and RadB from *Pyrococcus furiosus* have been shown to interact *in vitro* [17]. Therefore, RadB has been suggested to play a role in promoting HR, similar to the yeast Rad51 paralogue Rad55-57.

In this study, we elucidate the role of *H. volcanii* RadB in HR. We show that RadA and RadB interact *in vivo*, confirming previous *in vitro* results. We show that RadB is required for normal cellular growth, efficient HR and survival following treatment with DNA-damaging agents. Most significantly, we identify two point mutations in *radA* that suppress the Δ*radB* phenotype. The location and identity of these two amino acid substitutions leads us to propose that RadB induces a conformational change in RadA and thereby promotes its polymerisation on DNA.

## 2 Materials and methods

### 2.1 Strains and plasmids

*Haloferax volcanii* strains are shown in Table 1, plasmids used for gene deletion and protein overexpression in Table 2 and oligonucleotides in Table 3. Growth and transformation of *H. volcanii,* isolation of genomic and plasmid DNA, and construction of deletion mutants was carried out as described [18]. Protein over-expression strains were constructed by transformation with episomal overexpression plasmids as described [19]. Strains expressing tagged proteins at native levels were constructed by gene replacement as described [18].

**Table 1:** *H. volcanii* strains.

Strain	Genotype	Derivation	Use
H26	*ΔpyrE2*	[18]	Standard laboratory strain
H64	*ΔpyrE2 radBΔb/b*	H26 pTA62	Partial deletion of *radB*
H187	*ΔpyrE2 radBΔb/b Δhjc*	H64 *Δhjc*	*hjc* deletion in *radBΔb/b* background
H188	*ΔpyrE2 radBΔb/b Δhjc radA-A196V*	H187 *radA-A196V*	Spontaneous *radA-A196V* in *radBΔb/b* background
H195	*ΔpyrE2 ΔhdrB ΔtrpA bgaHa-Bb leuB-Ag1*	[16]	Background for recombination assays
H284	*ΔpyrE2 ΔhdrB ΔtrpA bgaHa-Bb leuB-Ag1 ΔradB*	[16]	*radB* deletion strain
H388	*ΔpyrE2 ΔhdrB ΔtrpA bgaHa-Bb leuB-Ag1 ΔradA:trpA+*	H195 pTA324	*radA* deletion, pTA411 also used
H724	*ΔpyrE2 ΔhdrB ΔtrpA bgaHa-Bb leuB-Ag1 ΔradB radA-A196V*	H284 pTA769	*radA-A196V* in a *ΔradB* background
H769	*ΔpyrE2 ΔhdrB ΔtrpA bgaHa-Bb leuB-Ag1 radA-A196V*	H724 pTA311	*radA-A196V* strain
H1309	*ΔpyrE2 radBΔb/b radA-S101P*	H64 EMS	EMS-induced *radA-S101P* in *radBΔb/b* background
H1424	*ΔpyrE2 ΔhdrB Δmrr Nph-pit cdc48-Ct*	[26]	Background for protein expression
H1428	*ΔpyrE2 ΔhdrB ΔtrpA bgaHa-Bb leuB-Ag1 ΔradB radA-S101P*	H284 pTA1289	*radA-S101P* in *ΔradB* background
H1439	*ΔpyrE2 ΔhdrB ΔtrpA bgaHa-Bb leuB-Ag1 radA-S101P*	H195 pTA1289	*radA-S101P* strain
H1450	*ΔpyrE2 ΔhdrB Δmrr Nph-pit cdc48-Ct* *{p.tnaA:his6tag-radB+ pyrE2+ hdrB+}*	H1424 pTA1043	Overexpression of His-tagged RadB
H1466	*ΔpyrE2 ΔhdrB ΔtrpA bgaHa-Bb leuB-Ag1 ΔradB* *radA+:[radA-S101P-A196V pyrE2+]*	H284 pTA1314	Integration of pTA1314, *radA-S101P-A196V* not viable
H1681	*ΔpyrE2 ΔhdrB ΔtrpA bgaHa-Bb leuB-Ag1 ΔradB ΔradA:trpA+*	H284 pTA324	*radA radB* deletion, pTA411 also used
H2047	*ΔpyrE2 ΔtrpA Δmrr Nph-pit cdc48-Ct*	H1424 pTA95 [18]	Protein expression strain, *ΔtrpA*
H2378	*ΔpyrE2 ΔtrpA Δmrr Nph-pit cdc48-Ct* *ΔradB:trpA+*	H2047 pTA1539	*radB* deletion in protein expression strain
H3041	*ΔpyrE2 ΔtrpA Δmrr Nph-pit cdc48-Ct* *his7tag-2xStrepIItag-radB+*	H2378 pTA1847	Expression of His-tagged RadB at native level
H3117	*ΔpyrE2 ΔhdrB ΔtrpA bgaHa-Bb leuB-Ag1 ΔradB* *radA+:[radA-S101A pyrE2+]*	H284 pTA1868	Integration of pTA1868, *radA-S101A* not viable in *ΔradB* background
H3231	*ΔpyrE2 ΔhdrB ΔtrpA bgaHa-Bb leuB-Ag1 radA-S101A*	H195 pTA1868	*radA-S101A* strain
H3264	*ΔpyrE2 ΔhdrB ΔtrpA bgaHa-Bb leuB-Ag1 radA-S101A* *radB+:[ΔradB:trpA+ pyrE2+]*	H3231 pTA1539	Integration of pTA1539, *ΔradB* not viable in *radA-S101A* background

**Table 2:** Plasmids.

Plasmid	Relevant properties	Derivation
pGB70	Integrative plasmid based on pUC19, with *pyrE2* marker	[36]
pTA50	pBluescript II with Eco47III-XmaI chromosomal fragment containing *radB*	[16]
pTA62	pGB70 with *radBΔb/b* partial deletion, generated by excision of BstBI-BstEII fragment of *radB* from pTA50	This study
pTA131	Integrative plasmid based on pBluescript II, with *pyrE2* marker	[18]
pTA163	Integrative plasmid containing *leuB-Aa2* allele, for use in recombination assay	[20]
pTA289	pTA131 with *ΔradB* construct	[16]
pTA311	pTA131 with *radB^+^*, generated by insertion of KpnI-BspEI fragment of pTA50 containing *radB*	This study
pTA324	pTA131 with *ΔradA:trpA^+^* construct	[25]
pTA409	Shuttle vector based on pBluescript II, with *pyrE2* and *hdrB* markers and *ori-pHV1* origin	[25]
pTA411	pTA409 with *radA^+^* gene, for complementation of *ΔradA*	[25]
pTA769	pTA131 with *radA-A196V*, generated by PCR of KpnI-BstBI *radA-A196V* fragment from H188	This study
pTA963	Overexpression vector with *p.tnaA* promoter, 6xHis tag, *pyrE2* and *hdrB* markers, and pHV2 origin	[19]
pTA1043	pTA963 with *radB*, for overexpression of 6xHis-tagged RadB	[19]
pTA1289	pTA131 with *radA-S101P*, generated from pTA769 by replacement with AgeI-BstEII *radA-S101P* fragment from H1309	This study
pTA1314	pTA131 with *radA-S101P-A196V*, generated from pTA1289 by replacement with AflIII fragment from pTA769	This study
pTA1539	pTA131 with *ΔradB:trpA^+^* construct, generated by PCR of XhoI-BamHI fragment of upstream flanking region and BamHI-XbaI fragment of downstream flanking region from pTA50, with insertion of *trpA+* BamHI fragment of pTA298 [20]	This study
pTA1771	pTA131 with insertion of *his7tag-2xStrepIItag* cassette at EcoRV site in multiple cloning site, features NdeI site upstream of 7xHis tag and PciI site downstream of 2xStrepII tag	This study
pTA1815	pTA1771 with insertion of FatI-BamHI *radB^+^* fragment of pTA1043, at PciI and BamHI sites	This study
pTA1847	pTA1539 with replacement of *ΔradB:trpA^+^* by NdeI-BamHI fragment of pTA1815 with *his7tag-2xStrepIItag-radB+* allele	This study
pTA1868	pTA131 with *radA-S101A*, generated from pTA1289 by replacement with AgeI-BstEII PCR fragment using radAS101Aint primers	This study

**Table 3:** Oligonucleotides

Primer	Sequence (5′–3′)	Relevant properties	Plasmid
RADAF	GGggATCCGTGGGACTAACCGCGCTCGCCCGTCGTGCCTG	Amplification of *radA*	pTA769 pTA1289
RADAR	CGTCGGAtcCCAGCGTTACCCCCACGTCGCCGTCG	Amplification of *radA*	pTA769 pTA1289
pradAF	TATCGCCCTTGAATCTCCGCAC	Introduction of S101A point mutation in *radA*	pTA1868
pradARTF	GACGATACGCTTGTCGCCC	Introduction of S101A point mutation in *radA*	pTA1868
radAS101AintF	CGCAGgCgATCACCGAGGTGTACGG	Introduction of S101A point mutation in *radA*	pTA1868
radAS101AintR	GTGATcGcCTGCGTTTCGAGACGCG	Introduction of S101A point mutation in *radA*	pTA1868
dradBBamR	CGGTGGAtcCTGACTCTGTCACGTCAGG	*radB* deletion, upstream	pTA1539
dradBXhoF	CGGTCTCGagGCGGACCGTTAGGCAGCCG	*radB* deletion, upstream	pTA1539
dradBdsBamF	AAAAGGGaTCcACGCGGCCGGGGAGACG	*radB* deletion, downstream	pTA1539
dradBdsXbaR	CCGGTCTAgaAGGGCGAAAAACAGTACGG	*radB* deletion, downstream	pTA1539
7His2xStrepF	caTATGCACCACCACCACCACCACCACGGCACGTCGGGCTGGTCGCACCCGCAGTTCGAGAAGGGCGGCTCGGGCTGGTCGCACCCGCAGTTCGAGAAGGGCGGCGAcatgt	*his7tag-2xStrepIItag* cassette	pTA1771
7His2xStrepR	aCATGTCGCCGCCCTTCTCGAACTGCGGGTGCGACCAGCCCGAGCCGCCCTTCTCGAACTGCGGGTGCGACCAGCCCGACGTGCCGTGGTGGTGGTGGTGGTGGTGCAtatg	*his7tag-2xStrepIItag* cassette	pTA1771

### 2.2 Growth curves

Growth curves of 250 μl cultures were performed in 48-well plates at 45 °C, with continuous double-orbital shaking at 425 rpm, using a BioTek Epoch2 microplate spectrophotometer. Optical density at 600 nm was measured every 15 min. Generation time was calculated between A600 values of 0.08–0.16.

### 2.3 Recombination assays

Plasmid × chromosome recombination assays were carried out as described [20].

### 2.4 Bioinformatic analyses

Primer design, and DNA and protein sequence analysis were performed using MacVector (MacVector Inc.). Predicted hydrophobicity indices were calculated using the Kyte-Doolittle scale [21]. Sequence alignments were performed using ClustalW [22] (Gonnet Series, open gap penalty of 10.0, extended gap penalty of 0.2, Delay Divergent value of 30%). *Pfu* RadA protein structure (1PZN) was obtained from Protein Data Bank (www.rcsb.org/pdb) and analysed using MacPyMOL (DeLano Scientific) [23].

### 2.5 Random mutagenesis

EMS (ethyl methane sulphonate, Sigma) was used for random mutagenesis as described [24] with the following modifications. Strains were grown in Hv-YPC broth to an A650 of 0.2, EMS was added to 3.5 μl/ml and mixed by gentle vortexing. Cells were incubated for 2 h at 45° C with rotation, washed twice with 18% salt water and resuspended in 1 ml of Hv-YPC broth. The culture was incubated overnight at 45° C with rotation, followed by plating on Hv-YPC agar. Plates were incubated for 5 days.

### 2.6 DNA damage assays

Assays for sensitivity to UV light and mitomycin C were carried out as described [20], [25].

### 2.7 Protein overexpression and purification

Protein (over)-expression and purification by metal-affinity chromatography (IMAC) was carried out as described previously [26] with the following modifications: cells were resuspended in buffer (2 M NaCl, 20 mM HEPES pH 7.5, 20 mM imidazole) containing 1× SigmaFAST protease inhibitor (Sigma) in replacement of 1 mM phenylmethanesulfonyl fluoride; lysate was incubated with the Ni^2+^ charged beads for 1 h at 4 °C; bound proteins were eluted in 4 column volumes (CV) of buffer containing 100 mM imidazole in place of 500 mM imidazole.

### 2.8 Mass spectrometry

Mass spectrometry of excised protein bands was carried out as described [19]. Details of protein identification are given in Supplementary Tables 1 and 2.

## 3 Results

### 3.1 RadA and RadB interact *in vivo*

**Fig. 1:**
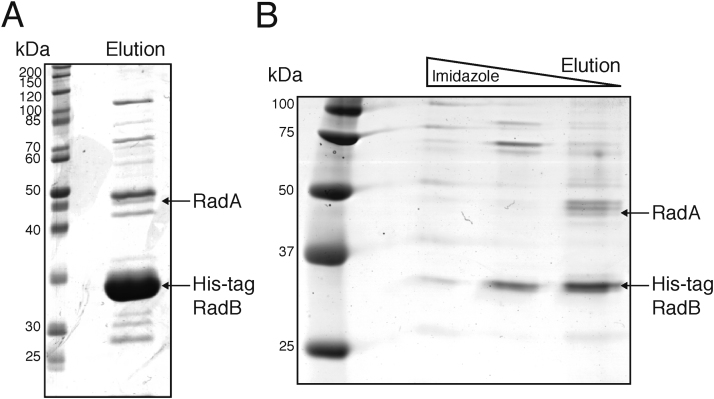
(A) RadA co-purifies with His-tagged RadB, which was over-expressed in *H. volcanii* (H1450) and purified by metal affinity chromatography (IMAC). Other proteins identified also purified from the parental strain H1424 containing an empty vector, which was used as a control for non-specific binding to the IMAC column [26]. (B) RadA also co-purifies with His-tagged RadB expressed in *H. volcanii* (H3041) at native levels. For mass spectrometry data, see Supplementary Tables 1 and 2.

RadB from *P. furiosus* has been shown by co-immunoprecipitation to interact with RadA *in vitro* [17]. To test whether RadB and RadA from *H. volcanii* interact *in vivo*, cell lysate from strains over-expressing His-tagged RadB (or RadA) was purified by metal-affinity chromatography (IMAC). Tagged and co-purifying proteins were eluted and analysed by SDS-PAGE, and bands of interest identified by mass spectrometry (Supplementary Table 1). In agreement with previous studies, RadA was found to co-purify with His-tagged RadB (Fig. 1A). RadB was not found to co-purify with His-tagged RadA (data not shown), but intracellular levels of RadB are known to be low; in *P. furiosus*, levels of RadB have been shown to be approximately 200 times lower than the levels of RadA [17]. Therefore, the method used here may not be sensitive enough [17].

To validate this interaction, a strain was generated where His-tagged RadB is expressed at native levels. A *radB* allele encoding His-tagged RadB was placed under control of the *radB* promoter and used to replace the wild-type (untagged) *radB* at its chromosomal locus. RadA was found by mass spectrometry to co-purify with natively-expressed His-tagged RadB, which had been purified by IMAC (Fig. 1B, Supplementary Table 2). Therefore, RadA and RadB interact *in vivo*, suggesting that they function together.

### 3.2 RadB is required for efficient DNA repair by HR

It has been shown that strains deleted for *radA* are completely deficient in recombination [4]. We examined the effect of *radB* deletion by carrying out a plasmid × chromosome recombination assay (Supplementary Fig. S1). The Δ*radB* mutant exhibited a recombination frequency of approximately 1.8% of wild-type (3.39 × 10^−6^ vs. 2.97 × 10^−4^ transformants per μg DNA per cell, respectively). Therefore, RadB is not essential for recombination (in contrast to RadA) but its presence dramatically improves the efficiency of this process.

**Fig. 2:**
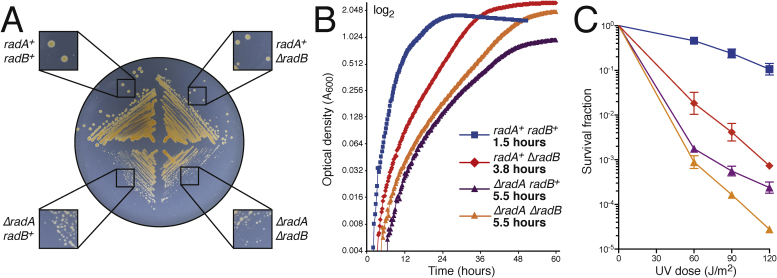
(A) Both Δ*radA* (H388) and Δ*radB* (H284) strains have a growth defect compared to wild-type (H195). The double mutant (H1681) has a similar growth defect to the *ΔradA* mutant. (B) The growth defect of *ΔradA* and *ΔradB* strains is also seen in broth; the double mutant has the same growth defect as the *ΔradA* mutant. Data was plotted on a log_2_ scale, generation time in exponential phase is shown in bold. (C) Both Δ*radB* and Δ*radA* strains are more sensitive to UV-irradiation than wild-type. The double mutant shows a similar sensitivity to the *ΔradA* mutant. Survival is relative to an unirradiated control. Each data point is an average of ≥3 independent repeats; standard error is shown.

We have previously shown that strains deleted for *radB* have a growth defect and are sensitive to UV radiation [16]. Strains deleted for *radA* also show a growth defect and DNA-damage sensitivity [4]. To study the relationship between RadA and RadB, a Δ*radA* Δ*radB* strain was generated. We confirmed that strains deleted for either *radA* or *radB* have a growth defect (compared to wild-type), and that Δ*radA* strains have a more severe defect (Fig. 2A and B). The Δ*radA* Δ*radB* double mutant shows a similar growth defect to the *ΔradA* single mutant, therefore *radA* is epistatic to *radB*. This suggests that with respect to cellular growth, the primary role of RadB is in HR.

Strains deleted for *radA* or *radB* have been shown to be sensitive to DNA-damaging agents [4], [16]. The Δ*radA* Δ*radB* double mutant was irradiated with UV light and its sensitivity compared to the single mutants (Fig. 2C). The Δ*radA* strain is more sensitive than the *ΔradB* strain, and at higher UV doses the *ΔradA ΔradB* strain is slightly more sensitive than the *ΔradA* mutant. This suggests that with respect to the repair of UV-induced lesions, RadB acts primarily in HR but may play a minor secondary role in another repair pathway.

### 3.3 Isolation of ΔradB suppressors

**Fig. 3:**
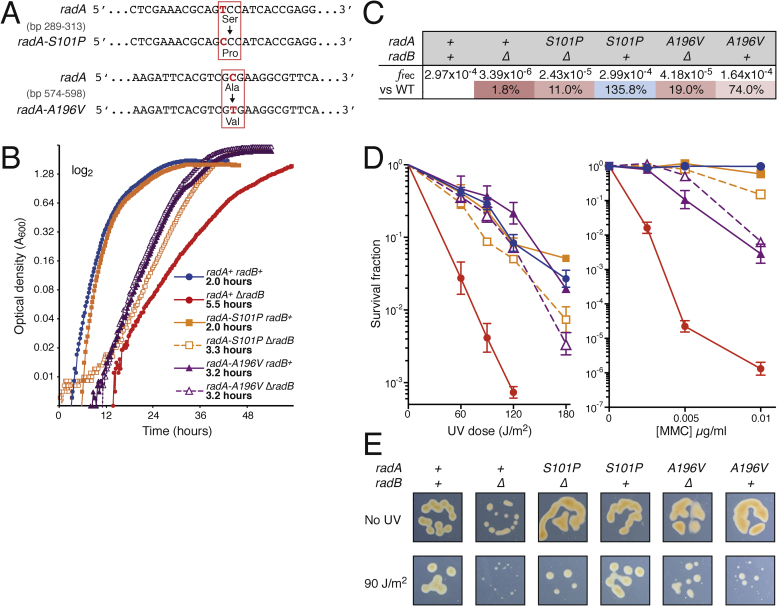
(A) Base substitutions in *radA* that result in *radA-S101P* and *radA-A196V*. (B) Δ*radB* strains have a growth defect in broth compared to wild-type, and *radA-S101P* (H1428) or *radA-A196V* (H724) suppress this defect. Data was plotted as in Fig. 2B. (C) Both *radA-S101P* and *radA-A196V* alleviate the recombination defect of *ΔradB* strains. Recombination frequency (*f*rec) was measured using the assay shown in Supplemental Fig. S1. Transformants per μg DNA per cell was calculated as an average of ≥3 independent repeats; percentages indicate recombination frequency compared to wild-type. D) Both *radA-S101P* and *radA-A196V* suppress the DNA damage defect of Δ*radB*. Survival following DNA damage (UV, left. MMC, right) is calculated relative to an unirradiated control, see panel B for key. Each data point is an average of ≥3 independent repeats; standard error is shown. E) Strains expressing *radA*-*A196V* recover more slowly than strains expressing *radA*-*S101P* after UV-irradiation. Cultures were spotted onto complete media and treated with 90 J/m^2^ of UV (or no UV as a control); colony size was observed after 5 days. All spots are 10^−5^ dilution except for the irradiated Δ*radB*, which is 10^−2^.

A significant insight into the role of RadB was gained from two point mutations that suppress the Δ*radB* phenotype; both are in the *radA* gene. The first of these to be identified, *radA-A196V*, was isolated as a spontaneous mutant based on improved growth of a Δ*radB* parent. Sequencing revealed a single point mutation in *radA* (Fig. 3A), a cytosine to thymidine transition at nucleotide 588 that results in an alanine to a valine substitution at amino acid 196. This mutation was confirmed as the Δ*radB* suppressor by replacing wild-type *radA^+^* with *radA-A196V* in a ‘clean’ *ΔradB* background (Supplementary Fig. S2).

The second point mutation was isolated by treating a Δ*radB* parent with the mutagen ethylmethane sulphonate (EMS) and screening for faster-growing colonies. This point mutation is a thymidine to cytosine transition at nucleotide 301 that results in a serine to proline substitution at amino acid 101 of RadA (Fig. 3A). The Δ*radB* suppressor was confirmed by introducing the same point mutation into a ‘clean’ *ΔradB* background that had not been subjected to mutagenesis (Supplementary Fig. S2).

### 3.4 RadA-S101P and RadA-A196V suppress ΔradB to differing degrees

Suppression of Δ*radB* by either *radA-S101P* or *radA-A196V* was measured by growth rate, recombination rate, and survival following DNA damage. Both *radA-S101P* and *radA-A196V* alleviate the growth defect associated with Δ*radB* to a considerable degree (Fig. 3B). The generation times of the *ΔradB radA-S101P* and *ΔradB radA-A196V* strains were 3.3 and 3.2 h, respectively, which is a marked improvement on *ΔradB* (5.5 h) but not as fast as wild-type (2.0 h). The presence of RadB in the *radB^+^ radA-A196V* strain did not lead to any further improvement in growth, but in the *radB^+^ radA-S101P* strain the presence of RadB restored the generation time to wild-type levels (2.0 h).

Both *radA-S101P* and *radA-A196V* alleles suppress the recombination defect associated with *ΔradB* (Fig. 3C), but the recombination frequencies were still lower than those seen in wild-type (11% of wild-type for *radA-S101P* and 19% for *radA-A196V*). For both alleles, the presence of RadB elevated the recombination frequency. Strains expressing both RadA-S101P and RadB have a recombination frequency above that of wild-type (135.8%), but in strains expressing RadA-A196V and RadB the level is below wild-type (74%). Therefore, *radA*-*S101P* and *radA*-*A196V* alleviate the recombination defect associated with Δ*radB* to differing extents.

Following irradiation with UV light, both *radA-S101P* and *radA-A196V* alleviate the DNA damage sensitivity conferred by Δ*radB* (Fig. 3D), and this was to the same extent for both mutations. When RadB is present in combination with these alleles, the UV-sensitivity was comparable to wild-type. After treatment with mitomycin C (MMC, DNA crosslinking agent), both *radA-S101P* and *radA-A196V* alleviate the DNA damage sensitivity conferred by Δ*radB* (Fig. 3D). In contrast to UV, survival of *ΔradB* strains after MMC treatment differ between the *radA-S101P* and *radA-A196V* alleles; these differences are also seen when RadB is present.

We noticed when monitoring UV sensitivity that *ΔradB* colonies are substantially smaller than those of unirradiated controls (Fig. 3E), indicating that the recovery of UV survivors is delayed. A delayed recovery from UV-induced damage was also seen in *ΔradB* strains expressing RadA-S101P or RadA-A196V. Strains expressing both RadA-A196V and RadB showed a greater delay in UV recovery than *ΔradB radA-A196V* strains, but this was not seen in strains expressing both RadA-S101P and RadB.

The suppression conferred by *radA-S101P* could be due to the presence of proline or the absence of serine (the latter is a common site of post-translational modification). To distinguish these possibilities, we attempted to generate a *ΔradB* strain containing a *radA-S101A* allele. This strain had a severe growth defect (worse than Δ*radA*) and could not be propagated. However, in a background containing wild-type RadB, the *radA-S101A* allele did not confer a growth defect. This indicates the absence of serine at residue 101 of RadA cannot suppress the growth defect associated with *ΔradB* (in fact, alanine it makes it worse), and suggests that the presence of a proline is most likely critical for suppression.

We also attempted to generate a *ΔradB* strain combining both *radA-S101P* and *radA-A196V* alleles. However, this strain had a severe growth defect (worse than Δ*radA*) and could not be propagated. This indicates that the presence of RadA-S101P-A196V is more detrimental than the complete absence of RadA.

### 3.5 Structural consequences of RadA-S101P and RadA-A196V

**Fig. 4:**
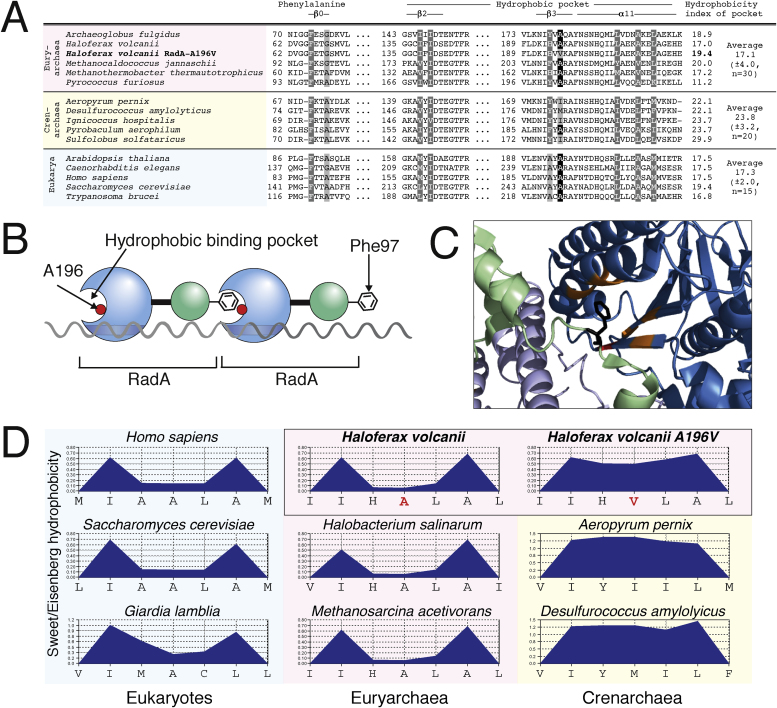
(A) Sequence alignment of the region of RadA/Rad51 containing the seven residues that comprise the hydrophobic socket (grey); shown are euryarchaeal (pink), crenarchaeal (yellow) and eukaryotic (blue) species. The residue equivalent to *H. volcanii* A196 is highlighted in black, it is conserved in euryarchaea and eukaryotes but not in crenarchaea. (B) RadA monomers polymerise by insertion of an invariant phenylalanine into a hydrophobic pocket of an adjacent monomer [23]. (C) Crystal structure of *Pyrococcus furiosus* (Pfu) RadA showing two RadA monomers (light green/blue and dark green/blue, respectively). Shown are the (core) ATPase domain (blue), N-terminal domain (green), hydrophobic binding pocket (orange), Pfu Ala203 (Hvo Ala 196) (red) and Pfu Phe97 (Hvo Phe66) (black). Crystal structure obtained from PDB (1PZN) [23]. D) Predicted hydrophobicity indices for the binding pocket of eukaryotic (blue), euryarchaeal (pink) and crenarchaeal (yellow) RadA/Rad51. Plots and overall average hydrophobicity were calculated using the Sweet/Eisenberg scale with a moving window of 3. The binding pocket of *H. volcanii* RadA-A196V (top row, right) has a higher predicted hydrophobicity than wild-type RadA (top row, centre), resembling the crenarchaeal binding pocket.

**Fig. 5:**
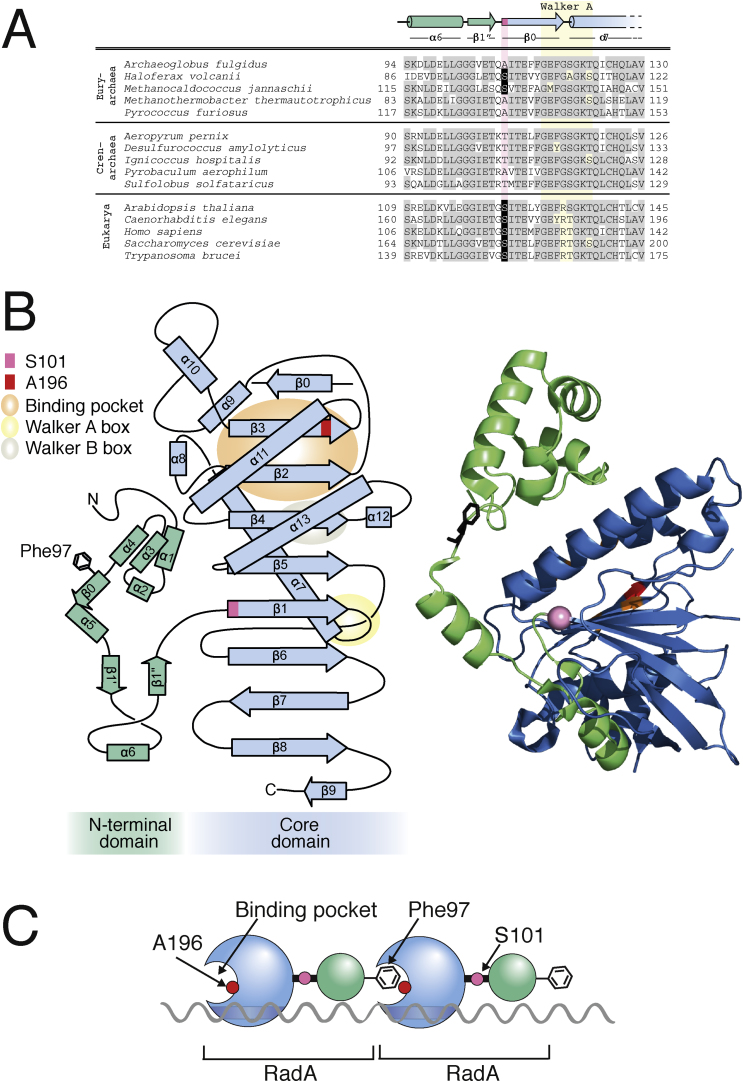
(A) *H. volcanii* RadA-S101 is conserved in euryarchaea and eukaryotes, but not crenarchaea. Sequence alignment of the RadA/Rad51 region containing *H. volcanii* RadA-S101. The equivalent residues for *H. volcanii* RadA-S101 are highlighted in pink and conserved serine highlighted in black. RadA from euryarchaea and crenarchaea, and Rad51 from eukaryotes are shown. Labelled at the top are the locations of conserved ß-sheets and α-helices [23]; the Walker A motif is shown in yellow. (B) Topology map (left) and crystal structure (right) of RadA monomer (adapted from Ref. [23]). The core domain is coloured blue and the N-terminal domain green. S101 is located at the joint between these two domains, just after a linker loop. (C) Schematic of RadA polymerisation via a ball and socket mechanism.

Amino acid residues corresponding to S101 and A196 in *H. volcanii* RadA are conserved in Euryarchaeota and eukaryotes, but not in Crenarchaeota. Since RadB is found only in Euryarchaeota (and not in Crenarchaeota), the conservation of *H. volcanii* RadA-S101 and A196 in archaea correlates with the presence of RadB. There is currently no crystal structure for *H. volcanii* RadA, therefore the corresponding residues were mapped onto *P. furiosus* RadA ([23], PDB number 1PZN). The equivalent residues to *H. volcanii* RadA-S101 and A196 in *P. furiosus* are RadA-A132 and A203, respectively (Figs. 4 A and 5 A ).

Archaeal RadA and eukaryotic Rad51 are conserved on a structural level. They consist of a core ATPase domain containing Walker A and B motifs for ATP binding and hydrolysis (respectively), and an N-terminal domain. Polymerisation occurs by the insertion of an invariant phenylalanine (Phe-96 in *H. volcanii*) located in the N-terminal domain of one monomer into a binding pocket of an adjacent monomer [23] (Fig. 4B). This binding pocket consists of seven surface-exposed hydrophobic residues (Fig. 4C), and monomer:monomer interactions are driven by hydrophobic interactions. Replacement of the invariant phenylalanine of human RAD51 with glutamic acid abolishes RAD51 polymerisation on ssDNA [27]. Hvo-RadA-A196 maps to the hydrophobic binding pocket implicated in RadA monomer:monomer interactions [23]. Valine and alanine are similar in size but valine is more hydrophobic. Substitution of valine for alanine in RadA-A196V increases the predicted hydrophobicity of the *H. volcanii* RadA pocket by 12% (Fig. 4D), which may result in stronger interactions between RadA monomers.

Hvo-RadA-S101 is located at the N-terminal end of the ß-1 sheet (Fig. 5B). This residue is at a joint between the two domains of RadA and this region has previously been described as an “elbow” [23]. Substitution of a proline in this position could induce a kink in this region of RadA – proline imposes constraints on the protein backbone and is commonly found in turns. This would alter the orientation of the N-terminal domain and may facilitate the polymerisation of RadA (Fig. 5C).

## 4 Discussion

### 4.1 RadA and RadB interact, and RadB acts in HR

By protein co-purification we show that *H. volcanii* RadA and RadB interact *in vivo* (Fig. 1). This is in agreement with previous observations in *P. furiosus* showing such an interaction *in vitro* [17]. As expected, RadB was found to play a critical role in HR. Deletion of *radB* leads to growth defects and sensitivity to DNA damage (Fig. 2), and reduces the level of recombination to 1.8% of wild-type. But in contrast to RadA [4], [16], RadB is not essential for HR. Therefore, RadA is able to carry out strand exchange by itself, but with greatly reduced efficiency. This supports the hypothesis that RadB functions as a recombination mediator [15].

The double Δ*radA* Δ*radB* mutant is slightly more sensitive to UV radiation than either single mutant. This synthetic defect suggests that RadB plays an additional role in DNA repair. Due to the complete abolition of HR in a Δ*radA* strain, it can be inferred that this additional role is not in recombination. In *P. furiosus*, RadB interacts with PolD1, the small subunit of DNA polymerase D [28] and in Pyrococcales, *radB* is located in an operon with *polD1*. Perhaps RadB plays a minor role in DNA replication.

### 4.2 radA-S101P and radA-A196V suppress ΔradB

Two suppressors of Δ*radB* were isolated. Both *radA-S101P* and *radA-A196V* mutations alleviate the Δ*radB* phenotype in terms of growth, recombination and DNA repair (Fig. 3). Since the extent of suppression differs between the two alleles, we propose that they act in different ways. For example, *radA-S101P* alleviates the *ΔradB* growth defect to a greater extent than *radA-A196V*. Both mutations suppress the UV sensitivity of a *ΔradB* strain equally, but there are minor differences in survival following MMC treatment. This suggests a difference in the ability of the RadA variants to repair inter-strand DNA crosslinks, or to restart stalled replication forks. Both mutations alleviate the recombination defect of a Δ*radB* strain, although only to 11% and 19% of wild-type (*radA-S101P* and *radA-A196V*, respectively). Since the growth rate of the *ΔradB radA-S101P* mutant is identical to wild-type, this suggests that only a limited level of HR is required for normal cellular growth.

### 4.3 Model for suppression by mutant RadA

The two suppressor mutations are located in different regions of RadA (Fig. 5) and we propose that they act in different ways. Hvo-RadA-S101 is located at an “elbow” between the core ATPase domain and the N-terminal domain of RadA [23]. Substituting a proline for serine in RadA-S101P could induce a kink in the “elbow” region of RadA, altering the orientation of the N-terminal domain and thereby facilitating the polymerisation of RadA monomers. By contrast, substitution of alanine for serine in RadA-S101A was only possible in a strain containing RadB. This suggests that RadB stimulates HR by altering the conformation of RadA, and that RadA-S101P is already in an active conformation for polymerisation (Fig. 5); conversely, RadA-S101A is in a conformation that is refractory to polymerisation.

**Fig. 6:**
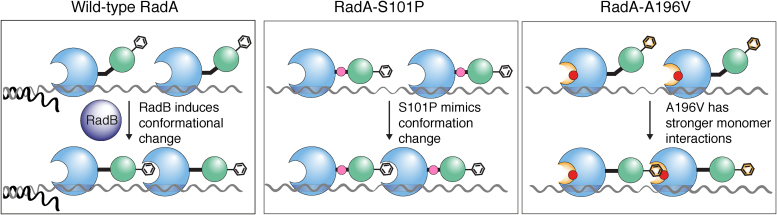
Model for RadB action. Wild-type RadA is not in the correct conformation for polymerisation and RadB is required. RadA-S101P is already in the correct conformation for polymerisation and does not require RadB. RadA-A196V has stronger hydrophobic interactions between monomers and does not require RadB.

Hvo-RadA-A196 maps to the hydrophobic binding pocket implicated in RadA polymerisation, which is driven by hydrophobic interactions [23]. Substitution of valine for alanine in RadA-A196V increases the predicted hydrophobicity of the RadA pocket, which is likely to result in stronger RadA:RadA interactions. This suggests that RadB plays a role in stabilising RadA filaments, and that RadA-A196V no longer requires RadB due to greater filament stability (Fig. 6). It is noteworthy that euryarchaea and eukaryotes have a similar predicted hydrophobicity of their RadA/Rad51 binding pocket (Fig. 4D), but crenarchaea, which do not have RadB, have a higher predicted hydrophobicity. This suggests that recombination mediators in eukaryotes and euryarchaea, such as RadB, may have a common mode of action.

If the two suppressors act in different ways, then a synergistic effect would be expected if they were combined. This would explain why we were unable to propagate a strain expressing *radA-S101P-A196V*. If RadA-S101P is already in an active conformation and RadA-A196V has stronger monomer:monomer interactions, then RadA-S101P-A196V filaments would polymerise rapidly due to S101P and be very stable due to A196V. This would result in slower dissociation from ssDNA, blocking the downstream processing of recombination intermediates. Kim et al. have recently shown that mutations that increase the recombination capacity of *E. coli* RecA have a detrimental effect on cellular growth [29]. This is due to the variant RecA filaments forming a barrier to replication and transcription.

### 4.4 Possible models for RadB activity

Previous studies have shown that mutant forms of the RecA-family recombinase can partially suppress defects associated with deletion of recombinase mediator genes, just as we have found for suppression of *ΔradB* defects by RadA-S101P or RadA-A196V. In *E. coli*, the mutant RecA730 protein can be loaded in the absence of RecFOR mediator because it is more proficient than the wild-type RecA in competition with SSB for ssDNA binding [30], [31]. In *Saccharomyces cerevisiae*, mutations in Rad51 that suppress the requirement for Rad55/57 mediators map to one of the DNA-binding sites of Rad51, thereby stabilising Rad51-DNA filaments and facilitating the displacement of RPA from ssDNA [32]. In both cases, the suppressor mutations improve ability of recombinase protein to bind DNA.

We propose that RadB induces a conformational change in RadA to facilitate efficient polymerisation. RadA polymerisation involves the insertion of an invariant phenylalanine into a hydrophobic socket of an adjacent monomer [23]. Based on our analysis of *H. volcanii* RadA-S101P, we propose that RadA monomers normally exist in an inactive form, where the phenylalanine is orientated away from the binding pocket of an adjacent monomer. RadB is required to alter the confirmation of RadA, thereby activating it (Fig. 6). Galkin et al. also proposed that the N-terminal domain of RadA/Rad51 undergoes a conformational change between the active (extended) and inactive (compressed) form, with only the extended form able to carry out strand exchange [33]. The authors propose that in the inactive form, the ATP binding site is rotated out of the filament, and that the N-terminal domain indirectly activates the filament by altering the conformation of the ATP binding site. By contrast, we propose that a conformation change directly activates RadA by relocating the phenylalanine in the correct position for polymerisation.

In naturally-competent bacteria, DprA binds cooperatively to ssDNA and activates RecA for nucleoprotein filament formation [34]. The ability to alter the conformation of recombinase filaments has also been observed for the recombination mediator RFS-1 from *C. elegans* [13]. Taylor et al. found that RFS-1/RIP-1 altered the conformation of RAD51, resulting in more flexible Rad51 filaments that facilitate the search for homologous sequences. RFS-1/RIP-1 does not appear to play a role in nucleating Rad51 filament formation in *C. elegans* (instead, BRCA2 plays this role). By contrast, we propose that RadB from *H. volcanii* can carry out this initial nucleation step. It is worth noting that no BRCA2 homologs or other recombination mediators have been identified in *H. volcanii* and therefore RadB may have evolved to perform multiple roles.

We expect RadA-A196V monomers to exist in a ‘wild-type’ conformation, since an alanine to a valine substitution in the binding pocket is unlikely to affect the structure of RadA. Instead, RadA-A196V is expected to have stronger hydrophobic monomer:monomer interactions (due to an increase in the hydrophobicity of the binding pocket), resulting in more stable RadA-A196V filaments. However, RadA filaments that are excessively stable would be unable to dissociate efficiently, and would block the downstream processing steps. This is consistent with our observation that strains expressing both RadA-A196V and RadB exhibit a delayed recovery from UV-irradiation, and suggests that RadB acts to stabilise RadA filaments. In yeast Rad55-Rad57 mediator complex has been shown to stabilise Rad51 filaments and counter the activity of the helicase Srs2 [12].

### 4.5 Comparison of genetic data on RadB with biochemical studies

Our genetic data shows that RadB is involved in HR and most probably assists in RadA polymerization. Biochemical evidence, however, does not necessarily support this hypothesis. RadB from *P. furiosus* was shown to inhibit RadA-mediated strand exchange [17] and the authors propose that this is due to RadB having a higher DNA binding affinity than RadA. We suggest an alternative explanation: *P. furiosus* RadB did not function as expected due to a missing protein co-factor. Eukaryotic recombination mediators such as human BRCA2 function as part of a multi-subunit complex [35], and *C. elegans* RFS-1 functions with its partner, RIP-1 [13]. Rad55 and Rad57 from *S. cerevisiae* function as a heterodimer, and Rad51 paralogues from higher eukaryotes function as heterodimers or tetramers.

We attempted to study the biochemical activity of wild-type and mutant *H. volcanii* RadA but were unable to purify RadA that is functional with respect to DNA binding and strand exchange (data not shown). This may be due to the difficulty of working with halophilic proteins, which require 2 M salt for activity. Alternatively, the reaction might be missing an essential protein co-factor. RadB was included in our strand exchange reactions but it might not be acting alone as a recombination mediator. To develop this study further, it will be necessary to identify all the interacting partner proteins of RadA and RadB in *H. volcanii*.

In conclusion, we provide evidence that RadB acts as a recombination mediator in *H. volcanii*. We propose that RadB induces a conformational change in RadA, allowing it to efficiently polymerise on ssDNA. Given the parallels between our findings and work in *C. elegans* [13], we expect that eukaryotic mediators might function in a similar manner.

## Conflict of interest statement

The authors declare that there are no conflicts of interest.

## Funding information

We are grateful to the Royal Society for a University Research Fellowship (516002.K5687) awarded to Thorsten Allers. This work was supported by the Wellcome Trust (grant number GR062124MF), and the Biotechnology and Biological Sciences Research Council (BBSRC) / Engineering and Physical Sciences Research Council (EPSRC) Synthetic Biology Research Centre Nottingham (grant number BB/L013940/1), through a PhD studentship awarded to Nathan Jones. The funders had no role in study design, data collection and interpretation, or the decision to submit the work for publication.
