# Supplementary figures and images for: RadB acts in homologous recombination in the archaeon Haloferax volcanii, consistent with a role as recombination mediator
Source: DNA Repair (Amst). 2017 Jul;55:7–16. doi: 10.1016/j.dnarep.2017.04.005 (PMC5480776; doi:10.1016/j.dnarep.2017.04.005)

# A

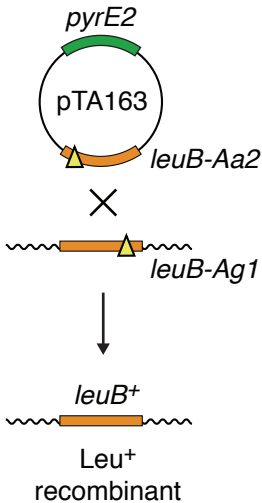

Supplement: Supplementary file 1 [file mmc1.pdf]
