# Supplementary material for: RadB acts in homologous recombination in the archaeon Haloferax volcanii, consistent with a role as recombination mediator
Source: DNA Repair (Amst). 2017 Jul;55:7–16. doi: 10.1016/j.dnarep.2017.04.005 (PMC5480776; doi:10.1016/j.dnarep.2017.04.005)
Supplement: Supplementary file 2 [file mmc2.pdf]

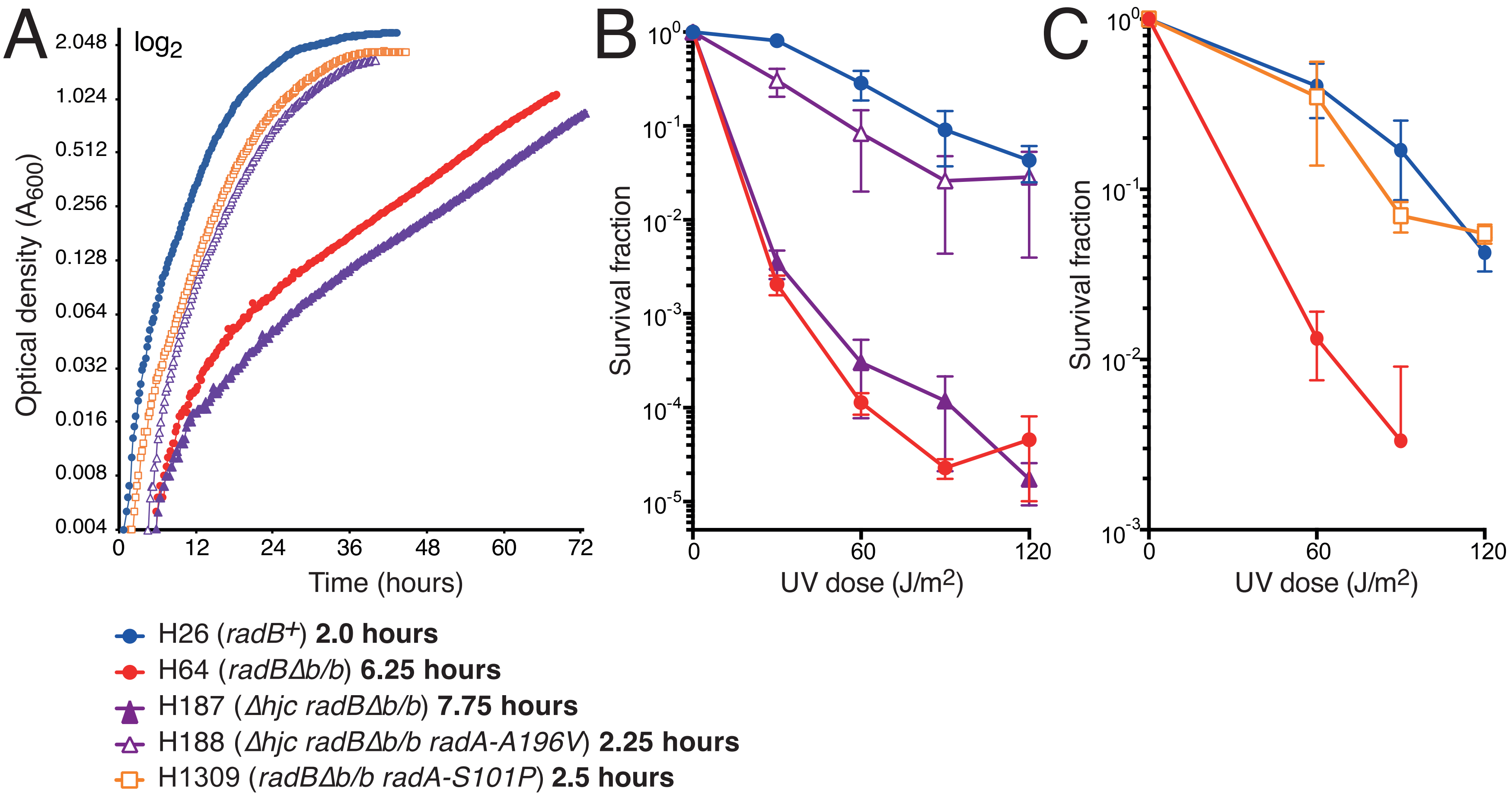
A
B
C
log2
Optical density (A600)
2.048
1.024
0.512
0.256
0.128
0.064
0.032
0.016
0.008
0.004
0
12
24
36
48
60
72
Time (hours)
Survival fraction
10^0
10^-1
10^-2
10^-3
10^-4
10^-5
0
60
120
UV dose (J/m2)
H26 (radB+) 2.0 hours
H64 (radBΔb/b) 6.25 hours
H187 (Δhjc radBΔb/b) 7.75 hours
H188 (Δhjc radBΔb/b radA-A196V) 2.25 hours
H1309 (radBΔb/b radA-S101P) 2.5 hours
