# Supplementary material for: RadB acts in homologous recombination in the archaeon Haloferax volcanii, consistent with a role as recombination mediator
Source: DNA Repair (Amst). 2017 Jul;55:7–16. doi: 10.1016/j.dnarep.2017.04.005 (PMC5480776; doi:10.1016/j.dnarep.2017.04.005)
Supplement: Supplementary file 3 [file mmc3.docx]

RadB Acts in Homologous Recombination in the Archaeon *Haloferax volcanii*, Consistent with a Role as Recombination Mediator

Kayleigh Wardell, Sam Haldenby, Nathan Jones, Susan Liddell, Greg H. P. Ngo, Thorsten Allers

**Supplementary Material**

## Supplementary Tables

***Table S1.*** Identification of relevant proteins present in cellular soluble fraction of *H. volcanii* H1450 over-expressing His-tagged RadB, after purification by affinity chromatography on a Ni^2+^ chelating column and excision from SDS-PAGE. Other proteins identified (not shown) were present in the empty vector control [[23](#_ENREF_23)].

| **Protein accession** | **Protein name** | **HVO_#** | **Predicted MW (kDa)** | **Observed MW (kDa)** | **MASCOT score** | **Number of peptides** | **Peptide sequences** | **Sequence coverage (%)** |
| --- | --- | --- | --- | --- | --- | --- | --- | --- |
| gi\|490144860 | RadA | HVO_0104 | 37.3 | 48 | 124 | 9 | 5 | 20 |
| gi\|292656510 | RadB | HVO_2383 | 25.5* | 33 | 1040 | 54 | 11 | 65 |

Protein Accession, database entry, e.g., gi|292656510; HVO_#, *H. volcanii* gene number; predicted MW, predicted molecular weight of protein sequence identified by MASCOT (*molecular weight of 6xHis-tagged RadB shown); observed MW, molecular weight estimated from migration on SDS-PAGE; MASCOT score, score associated with protein identification, searches were against Archaea entries of NCBInr (version 20150912); number of peptides, total peptides associated with protein identification; peptide sequences, number of distinct peptide sequences associated with identified protein; sequence coverage, percentage of database sequence entry covered by matched peptides.

***Table S2.*** Identification of relevant proteins present in cellular soluble fraction of *H. volcanii* H3041 expressing His-tagged RadB at native level.

| **Protein accession** | **Protein name** | **HVO_#** | **Predicted MW (kDa)** | **Observed MW (kDa)** | **MASCOT score** | **Number of peptides** | **Peptide sequences** | **Sequence coverage (%)** |
| --- | --- | --- | --- | --- | --- | --- | --- | --- |
| gi\|490144860 | RadA | HVO_0104 | 37.3 | 48 | 50 | 3 | 2 | 9 |
| gi\|292656510 | RadB | HVO_2383 | 28.6* | 33 | 280 | 22 | 7 | 44 |

*Molecular weight of 7xHis-2xStrepII-tagged RadB shown.

## Supplementary Figure Legends

**Figure S1**

Schematic for chromosome x plasmid recombination assay (adapted from [[17](#_ENREF_17)]). ∆*pyrE2* strains with a chromosomal *leuB*-*Ag1* allele (leu-) are transformed with the non-replicative plasmid pTA163, containing the *leuB*-*Aa2* allele. Recombination between the plasmid-borne *leuB-Aa2* allele and the chromosomal *leuB-Ag1* allele generates a wild-type *leuB^+^* gene, resulting in growth on media lacking leucine (Leu^+^). Recombination frequency per µg DNA (⨍_rec_) was calculated from the number of Leu^+^ colonies, as a fraction of viable cells. Viable cell count was determined by plating on Hv-YPC+thymidine; all strains had equal transformation frequencies (0.0025 per µg DNA, as a fraction of viable cells).

**Figure S2**

**A)** Growth in broth of *radB* deletion strains carrying suppressor mutations, which were isolated on the basis of faster growth (data was plotted as in Figure 2B). H188 was isolated as a spontaneous fast-growing mutant of H187, and H1309 was isolated as a fast-growing mutant of H64 following treatment with EMS; the wild-type H26 is shown for comparison. The *radA-A196V* suppressor mutation in H188 was isolated in a strain deleted for *hjc*, but subsequent analysis showed that Hjc plays no role in suppression of the *radB* phenotype (compare with Figure 3B and 3D). **B)** The fast-growing mutant H188 derived from *radB∆b/b ∆hjc* strain H187 does not exhibit the UV-sensitivity associated with deletion of *radB*; deletion of *hjc* has only a small effect on UV-sensitivity (H26 vs H187) [[17](#_ENREF_17)]. **C)** The fast-growing mutant H1309 derived from *radB∆b/b* strain H64 does not exhibit the UV-sensitivity associated with deletion of *radB*. Survival is relative to an unirradiated control. Each data point is an average of ≥3 independent repeats; standard error is shown.
